# Enriching public descriptions of marine phages using the Genomic Standards Consortium MIGS standard

**DOI:** 10.4056/sigs.621069

**Published:** 2011-04-29

**Authors:** Melissa Beth Duhaime, Renzo Kottmann, Dawn Field, Frank Oliver Glöckner

**Affiliations:** 1 Max Planck Institute for Marine Microbiology, Microbial Genomics, D-28359 Bremen, Germany; 2 Jacobs University Bremen gGmbH, D-28759 Bremen, Germany; 3 NERC Centre for Ecology and Hydrology, Maclean Building, Wallingford, Oxfordshire, OX10 8BB, United Kingdom; 4 University of Arizona, Tucson, Arizona, 85721, USA

**Keywords:** marine phages, contextual data, genome standards, markup language

## Abstract

In any sequencing project, the possible depth of comparative analysis is determined largely by the amount and quality of the accompanying contextual data. The structure, content, and storage of this contextual data should be standardized to ensure consistent coverage of all sequenced entities and facilitate comparisons. The Genomic Standards Consortium (GSC) has developed the “Minimum Information about Genome/Metagenome Sequences (MIGS/MIMS)” checklist for the description of genomes and here we annotate all 30 publicly available marine bacteriophage sequences  to the MIGS standard. These annotations build on existing International Nucleotide Sequence Database Collaboration (INSDC) records, and confirm, as expected that current submissions lack most MIGS fields. MIGS fields were manually curated from the literature and placed in XML format as specified by the Genomic Contextual Data Markup Language (GCDML). These “machine-readable” reports were then analyzed to highlight patterns describing this collection of genomes. Completed reports are provided in GCDML. This work represents one step towards the annotation of our complete collection of genome sequences and shows the utility of capturing richer metadata along with raw sequences.

## Introduction

Researchers interested in marine viruses have long acknowledged the need to link genomic data to both biogeochemical contextual data and host sequence data in order to maximally investigate marine virus-host systems [1]. Marine viruses contain a range of metabolically and environmentally significant genes, including those putatively involved in photosynthesis [2-4], nitrogen stress and vitamin biosynthesis [5], and nucleotide scavenging, thought to be a selective benefit in nutrient-poor open oceans [5,6].

The power to gain knowledge from any genomic venture depends heavily on the *a priori* sequence content of public databases with which to compare new sequences to, by sequence alignment approaches [7]. With nothing similar, new sequences can only be labeled as unknown, with no ‘handle’ by which to base functional or evolutionary hypotheses. The same ‘context-mining’ principle extends to sequence-associated contextual data. Sequences can be grouped by contextual parameters and then interpreted in a comparative context only when these data are available and stored in an accurate, structured and accessible fashion. This allows for interpretation in light of other organisms (or communities), including habitat, isolation location, biological features, the molecular procedures applied to obtain genomic material, sequencing and post-sequencing methods. Given the vast number of sequences already available, these contextual descriptors are becoming as valuable as the nucleotides that make up the sequences. When present and correct, the descriptors expand the number of dimensions available in the realm of comparative genomics and downstream hypothesis testing [8].

To promote better descriptions of our complete collection of genomes and metagenomes, the Genomic Standards Consortium (GSC) has published the “Minimum Information about a Genome/Metagenome Sequence” (MIGS/MIMS) checklist, which recommends a required set of contextual data, e.g., sample site latitude (*x*), longitude (*y*), depth (*z*), and time (*t*), to accompany all genomic sequence submissions to the public domain [9]. To facilitate the implementation of this standard, and promote the capture, exchange, and downstream comparison of MIGS contextual data, an XML schema has also been defined: the Genomic Contextual Data Markup Language (GCDML) [10].

Using the collection of sequenced marine phages as a case study, we have created a set of MIGS-compliant reports to (i) determine the effort required to make legacy data comply with the MIGS standard, (ii) determine the degree to which compliance is possible using public annotations and associated literature, and (iii) pave the way for the use of this information in exploratory analyses of marine phages.

## Methods

### Genomes and contextual data sources: MIGS-compliance

**Figure 1:**
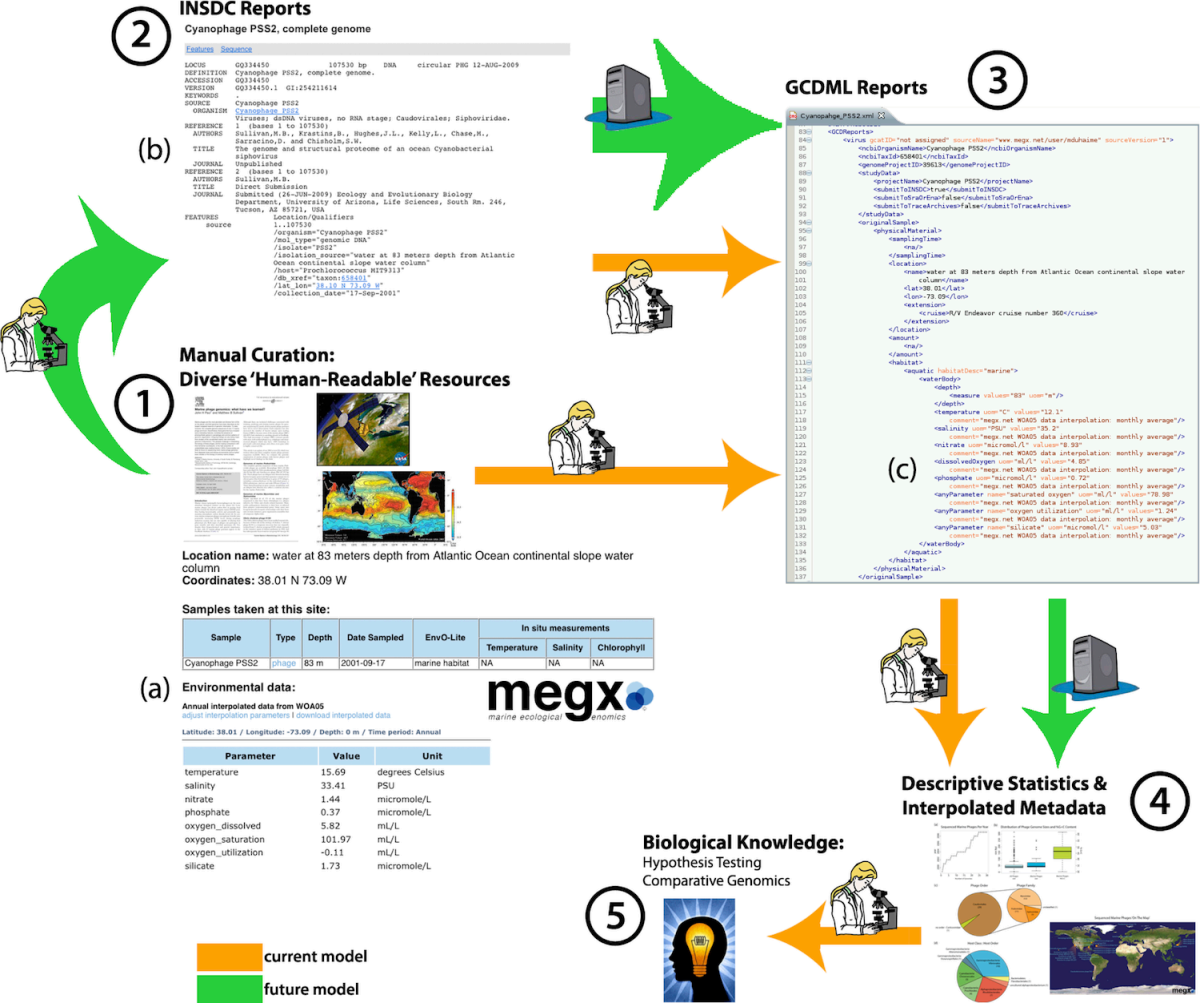
Model of flow of contextual data into biological knowledge. (a) screenshot of interpolated data for Cyanophage PSS2 from megx.net website (b) screenshot of Cyanophage PSS2 GenBank file, the only INSDC report to store x, y, z, t data, (c) section of GCD report showing GCDML structure, highlighting the storage of cruise information and interpolated data from megx.net GIS tools.

The complete set of phage genomes isolated from marine habitats was identified through literature [11] and text searches of PubMed. Associated genome files were collected in GenBank format (hereafter referred to as 'INSDC reports') along with publications describing the virus isolation and sequencing. Two datasets were then generated for comparison:

1. reports containing only MIGS fields available in the structured submitted INSDC reports (Panel 2 of Figure 1), and
2. manually created reports with complete MIGS information based on manual curation of diverse ‘human-readable’ resources (Panel 1 of Figure 1).

Manual curation required to complete the second set of files was significant (one to two months), as diverse resources were consulted. These included the literature, direct correspondence with authors, culture collections, and specialized databases, e.g., the Félix d'Hérelle Reference Center for Bacterial Viruses (FHRCBV), a highly curated reference catalog, which bases its taxonomy on morphology evident through their collection of high quality electron microscopy (EM) images of each phage [12]. Compliance with the ‘habitat’ descriptor of MIGS was achieved using terms from the EnvO-Lite (v1.4) controlled vocabulary [13]. Currently, INSDC reports do not explicitly define habitat as a field, however, when the INSDC location name contained a known marine habitat, the phage was labeled as ‘marine’ according to INSDC.

In addition, interpolated environmental parameters (temperature, salinity, nitrate, phosphate, dissolved oxygen, oxygen saturation, oxygen utilization, and silicate) describing the sampling sites were also assembled for all possible phage genomes (Table 1), using the megx.net GIS Tools [14]. This megx.net resource employs oceanographic data from large-scale datasets, such as the World Ocean Atlas [15], to interpolate data for single points in the oceans at one decimal degree of resolution [16].

**Table 1:** Phages, from a marine habitat, as reported in literature and their corresponding INSDC accession numbers.

**NCBI Organism Name**	**INSDC identifier**	**Interpolated data? ^[1]^**	**Missing Elements?**
Cyanophage PSS2	GQ334450	Yes	Complete
Flavobacterium phage 11b**^[2]^**	AJ842011	No - insufficient data	*x*, *y*, *z*, *t*
*Halomonas* phage phiHAP-1	EU399241	Yes	Complete
*Listonella* phage phiHSIC	AY772740	Yes	*x*, *y*
Phage phiJL001	AY576273	Yes	*x*, *y*
*Pseudoalteromonas* phage PM2	AF155037	No - insufficient data	*x*, *y*, *z*, *t*
*Prochlorococcus* phage P-SSP7	AY939843	Yes	Complete
*Prochlorococcus* phage P-SSM2	AY939844	Yes	Complete
*Prochlorococcus* phage P-SSM4	AY940168	Yes	Complete
*Roseobacter* phage SIO1	AF189021	No - insufficient data	*x*, *y*, *z*
*Roseobacter* phage SIO1-2001	FJ867910	No - insufficient data	*x*, *y*, *z*, *t*
*Roseobacter* phage SBRSIO67-2001	FJ867912	No - insufficient data	*x*, *y*, *z*, *t*
*Roseobacter* phage OS-2001	FJ867913	No - insufficient data	*x*, *y*, *z*, *t*
*Roseobacter* phage MB-2001	FJ867914	No - insufficient data	*x*, *y*, *z*, *t*
*Silicibacter* phage DSS3phi2	FJ591093	No - insufficient data	*x*, *y*
*Sulfitobacter* phage EE36phi1	FJ591094	No - insufficient data	*x*, *y*
*Synechococcus* phage P60	AF338467	No - insufficient data	*x*, *y*, *z*
*Synechococcus* phage S-PM2	AJ630128	No - insufficient data	*t*
*Synechococcus* phage S-RSM4	FM207411	No - insufficient data	*x*, *y*, *z*, *t*
*Synechococcus* phage syn9	DQ149023	No - too close to coast	x, y, t
*Synechococcus* phage Syn5	EF372997	Yes	*t*
*Vibrio* phage VP2	AY505112	No - insufficient data	*x*, *y*, *z*, *t*
*Vibrio* phage VP4	DQ029335	No - insufficient data	*x*, *y*, *z*, *t*
*Vibrio* phage VP5	AY510084	No - insufficient data	*x*, *y*, *z*, *t*
*Vibrio* phage VP16T	AY328852	No - too close to coast	*x*, *y*, *t*
*Vibrio* phage VP16C	AY328853	No -too close to coast	*x*, *y*, *t*
*Vibrio* phage VpV262	AY095314	No - insufficient data	*x*, *y*, *z*, *t*
*Vibrio* phage VHML **^[3]^**	AY133112	No - insufficient data	*x*, *y*, *z*, *t*
*Vibrio* phage KVP40	AY283928	No - insufficient data	*x*, *y*, *z*, *t*
*Vibrio* phage K139 **^[4]^**	AF125163	No - insufficient data	*x*, *y*, *z*, *t*

Phages finally determined *not* to be from marine habitats are noted in superscript and alternatively described according to EnvO-Lite (v1.4). Genomes for which interpolated data could be determined and missing elements required for geo-referencing are listed (note: *x*, *y*, *z* and tare required for *precise* metadata interpolation).

**1)** This can be as minimal as a “fuzzy” habitat descriptor (rather than precise *x*, *y*), requires a depth (or 'surface sample' description), and does not require a date (as yearly averages can be taken). However, if the sample site is too close to the shore, data interpolation is not possible.

**2)** isolated from sea ice (aquatic habitat)

**3)** isolated from aquacultured shrimp (organism-associated habitat)

**4)** isolated from human (organism-associated habitat)

## Generation of GCDML reports

**Figure 2:**
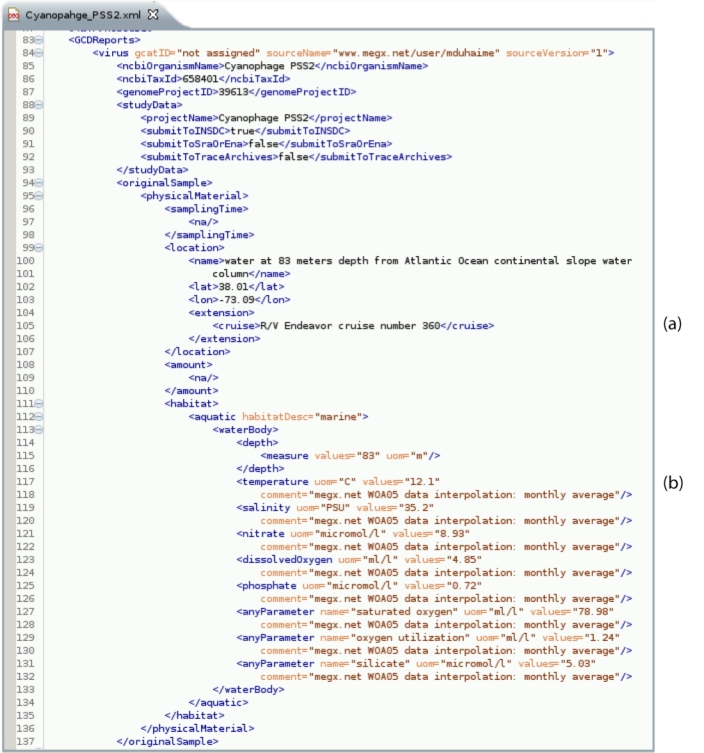
Screenshot GCDML Report revealing the GCDML schema using the Eclipse plug-in, oXygen. Note the (a) cruise data and (b) interpolated environmental parameters retrieved from megx.net for this genome can be added through the flexible GCDML ‘extensions.’

These curation efforts were used to inform early versions of GCDML. MIGS-compliant reports were rendered in GCDML, version 1.7 (Panel 3 of Figure 1, Figure 2) [10]. GCDML reports were manually created using the oXygen XML editor (version 11). Core MIGS fields were placed into GCDML and additional (optional) fields were placed into Genomic Contextual Data (GCD) reports (Panel 3c of Figure 1, Figure 2). These extensions allowed for consistent storage of genome size and %G+C content, latitude and longitude for ‘manually determined’ locations based on verbose geographic descriptors (rather than precise numeric reports), cruise ship name and number (allowing coordination with other samples collected on this cruise), and environmental metadata, either collected *in situ* or interpolated using, i.e., megx.net GIS tools (Panel 1a of Figure 1) [14]. All GCDML reports are available at the megx website [17].

## Exploratory contextual data analyses

Data describing all phages (size and taxonomy) were extracted from their respective GenBank files from NCBI (19 November 2009) with Perl scripts. A dendrogram clustering phages by sample site physical-chemical parameters (salinity, nitrate, dissolved oxygen, phosphate, oxygen saturation, oxygen utilization, and silicate) was derived from a distance matrix (Euclidean distance coefficient) of z-score transformed data using average linkage clustering. Phages were displayed on the megx.net map [16] using its integrated Web Map Service technology [16].

## Results and Discussion

### A comparison of INSDC reports and manually curated MIGS-compliant GCDML reports

**Figure 3:**
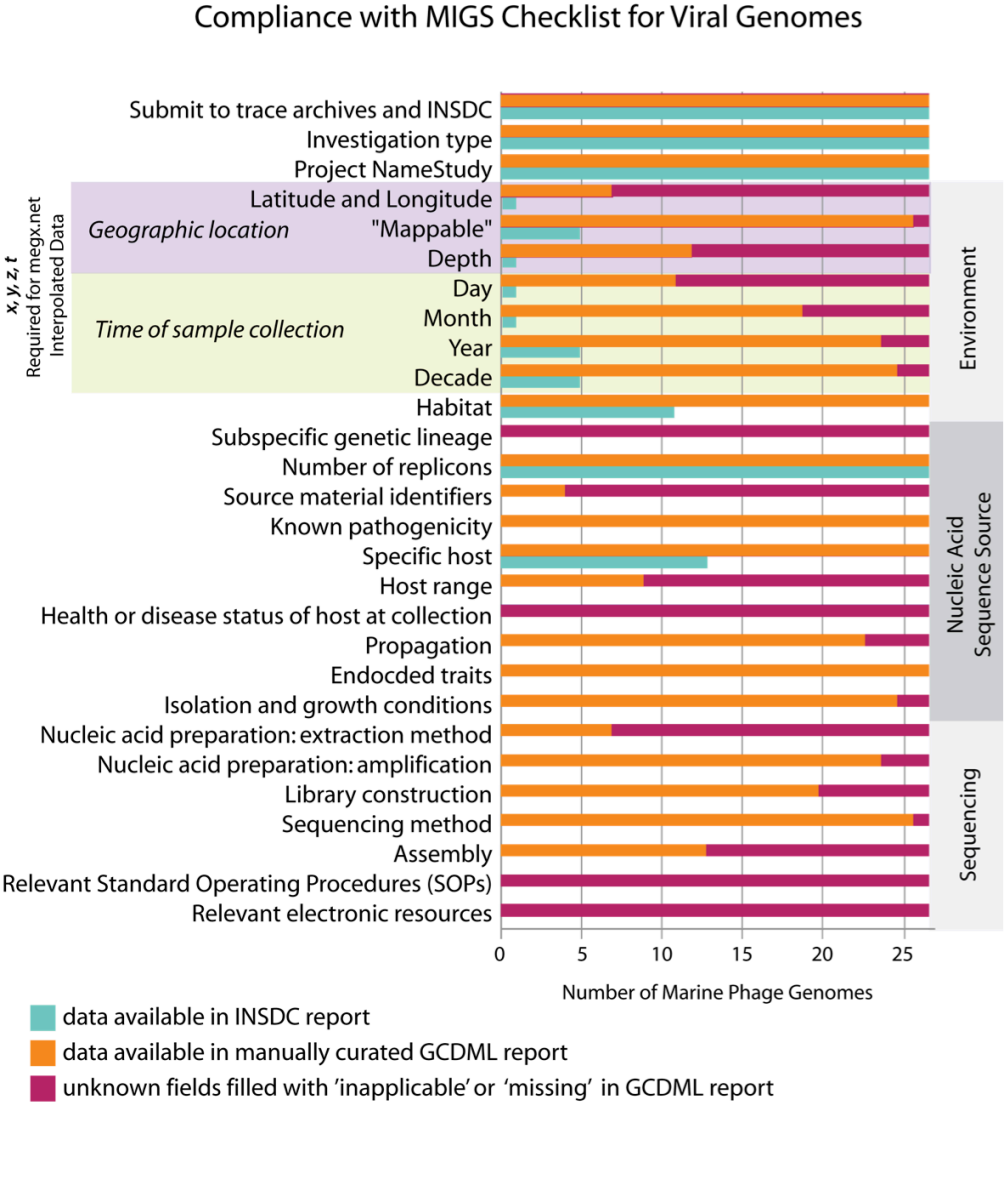
Comparison of compliance with viral components of the MIGS checklist between data available in INSDC reports and that in MIGS/GDC reports that have been supplemented with extensive manual curation. List modified from [9].

Surveying the literature and the public databases identified a set of 27 phages isolated from a ‘marine’ habitat (Table 1). Figure 3 compares the number of MIGS-compliant fields fulfilled by INSDC documents to those fulfilled after manual curation of the literature and other resources. Nearly half of the fields examined held no information in INSDC reports (especially pertaining to documentation of ‘Sequencing’ components), but following curation this rose to one hundred percent compliance (Figure 3). However, “unknown” (could not be determined) MIGS fields are filled with either an 'inapplicable' or ‘missing' qualifier, as this acknowledges the presence/absence of this information and therefore is more valuable than its complete absence from the report (Figure 3).

Overall, when the minimum required resolution of the field “date” is 'year', only 21% of the components recommended by the MIGS checklist are reported in the current marine phage INSDC reports (Figure 3). Through intensive manual curation it was possible to satisfy 66% of all MIGS components. Of the unknown components of the GCDML reports that still resisted manual curation (34%), one fourth are due to fields deemed 'inapplicable' for phages, such as 'Subspecific genetic lineage' and 'Health or disease status of host', both of which, though still components of the checklist, have been deemed not mandatory in the latest MIGS version, partly influenced by the experiences garnered in this study (unpublished update by GSC;[18]). The remaining three fourths of the fields are unknown due to missing information. Of the manually curated data, 1% of the fields could be confirmed only through personal communication with authors (e.g., to confirm habitat) or other experts in the field (e.g., to confirm taxonomy).

An essential piece of information about any genome is the habitat from which the genome (i.e., organism or sample) originated. To date, this information has not been captured systematically in public databases, yet is core to the MIGS specification due to its biological importance [19,20]. Information in INSDC reports made it possible to classify 41% of the phages as ‘marine’, meaning isolated from "A habitat that is in or on a sea or ocean containing high concentrations of dissolved salts and other total dissolved solids (typically >35 grams dissolved salts per litre)" (per Envo-Lite v1.4).

Following manual curation, three of the phages still could not be classified definitively as marine: *Vibrio* phage K139, *Vibrio* phage VHML, and Flavobacterium phage 11b (Table 1). The vibriophages are now annotated as ‘organism-associated’, having originated from "A habitat that is in or on a living thing" (per Envo-Lite v1.4). Kapfhammer *et al.* report that *Vibrio* phage K139 was isolated from its host lysogen, *Vibrio cholerae* O139 strain M010 [21], which is a clinical strain isolated in 1992 from the tenth *V. cholerae* O139 victim in Madras, India (Matthew Waldor, personal communication). *Vibrio* phage VHML was isolated from its host lysogen cultured from prawn larvae (*Penaeusmonodon*) from an aquaculture pond in Australia [22]. Flavobacterium phage 11b is now reported as ‘aquatic’, originating from "A habitat that is in or on water" (Envo-Lite v1.4). This phage was isolated from melted Arctic sea ice, a term which itself can not be classified as definitively marine, as sea ice has variable salinity depending on the ice growth stage or local structure, i.e., high-salinity brine chamber or low-salinity melt pool. In all, habitat curation (guided by an accepted habitat ontology) resulted in 27 'marine’ genomes, which are considered in the remaining analyses.

**Figure 4:**
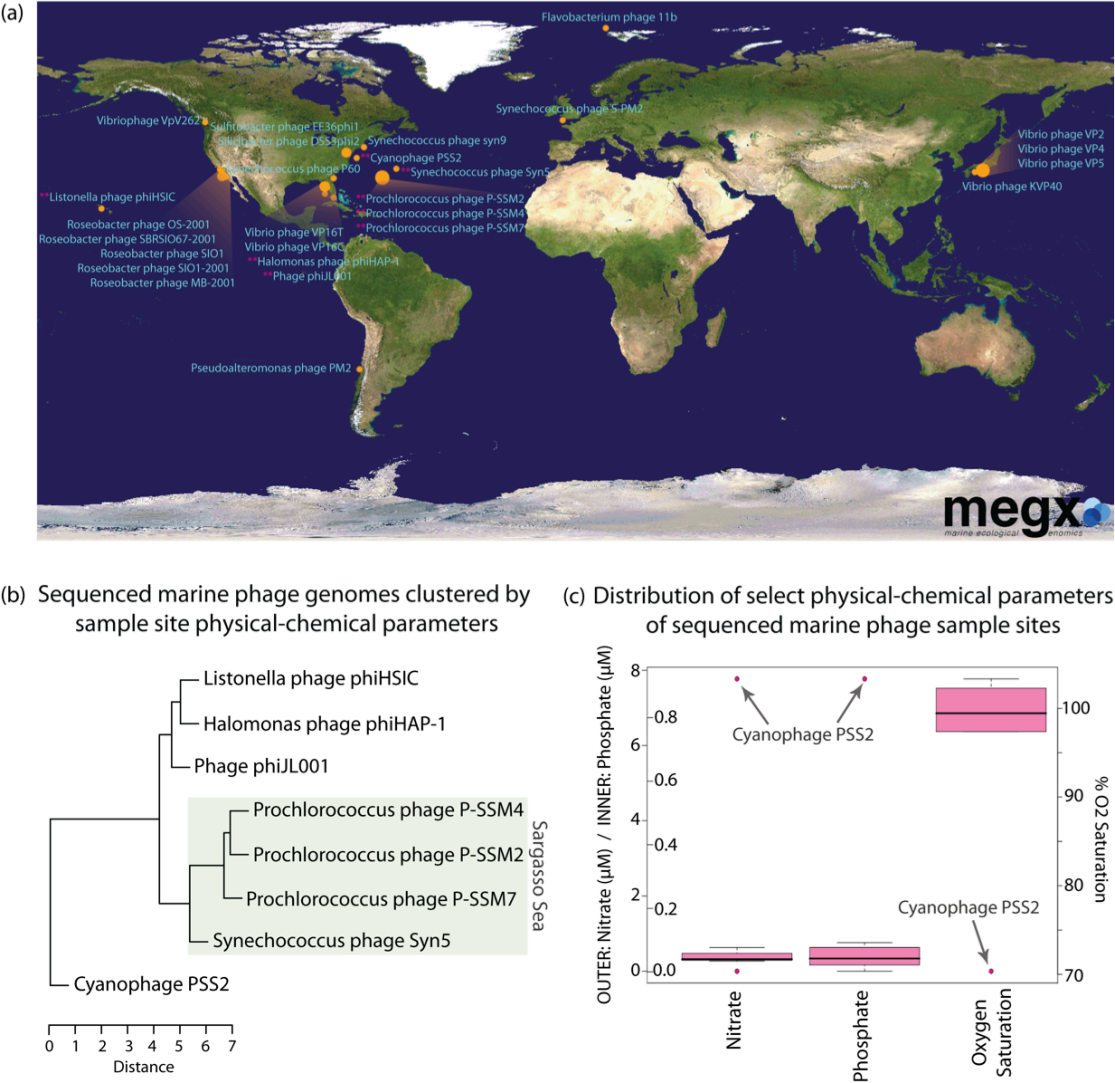
(a) The 26 'marine' phage genomes (plus 'aquatic' Flavobacterium phage 11b) able to be mapped based on data in their GCDML reports. The map is modified from that available from megx.net. See [23] for exact webserver query. For more information about the mapserver technology used by megx.net, see [24]; (b) sample sites of marine phages clustered by interpolated environmental data; (c) distribution of three of the interpolated environmental parameters (nitrate, phosphate, and oxygen saturation) demonstrating the Cyanophage PSS2 outlier.

Unsurprisingly [19,20], only a single marine phage, Cyanophage PSS2, contained sufficient latitude, longitude, and depth data (x, y, and z) in its INSDC report to place it conclusively on a map (Panel 2b of Figure 1; Figure 4). This was also the only INSDC report to contain depth. After manual curation, precise x and y coordinates were determined for only seven (26%) of the genomes. However, all but one phage (96%) were ‘mappable’, in that they described imprecise sample site descriptors, such as ‘Scripps Pier, La Jolla California, USA’ (Figures 2 and Figure 4). Depth could be added to 12 (44%); most manually curated depths were due to literature reports of “surface samples”, rather than exact depth measurements and reports. The union of x, y, z, and t (time) allows for extraction of interpolated environmental parameters; after manual curation, this data was available for only 11 (41%) of the phage genomes using megx.net GIS tools ([14]; Table 1). However, due to the inaccuracy of environmental data interpolation near land, the three sample sites too close to the coast are missing this data (Table 1).

Information on host-range and host taxonomy provides essential information on the biological and ecological impact of phages. INSDC reports stored information about host taxonomy in 48% of the reports. Information regarding host range was completely lacking from *all* INSDC reports. After manual curation, information about host taxonomy was expanded to 100% through manual curation (‘Specific Host’ Figure 3) and alternate hosts were manually determined for nine (33%) phages (‘Host Range’ Figure 3). The phage taxonomies documented in INSDC reports were compared to taxonomies documented in the phage isolation and sequencing publications, as well as to the Félix d'Hérelle Reference Center for Bacterial Viruses (FHRCBV). When conflicts occur, the FHRCBV is considered the expert taxonomy. For instance, *Vibrio* phage VP5 (NCBI taxid: 260827) is classified as *Podovirdae* in its INSDC report, whereas, according to the long non-contractile tail evident in the EM image in FHRCBV (accession: HER 169), it has been expertly classified as *Siphoviridae* (Sylvain Moineau, personal communication).

In addition to missing data, conflicting fields were also encountered. For example, the *Vibrio* phages VP2, VP4, and VP5, are reported as belonging to the *Podoviridae* in their INSDC genome reports. However, according to the Félix d'Hérelle Reference Center for Bacterial Viruses, VP5 belongs to the *Siphoviridae* (as confirmed by expert electron micrography), and VP2 and VP4 are described, with accompanying EM images, as myoviruses by Koga *et al*. in the description of their initial isolation [25]. Furthermore, the INSDC reports for *Vibrio* phages VP2, VP4, and VP5 report their host as *Vibrio cholerae*. This may be true for the phages used in the sequencing project in 2003 (though this can not be confirmed, as their genomes were directly submitted with no accompanying publication), however the phages were reportedly collected from seawater near Tokushima, Japan and isolated on *Vibrio parahaemolyticus* in 1982 [25].

## Exploratory Analysis

Contextual data is essential in gaining an understanding of the biology of these genomes as a group. Here we review key features of this collection of marine phage as highlighted by access to associated metadata, much of which is newly associated due to our manual curation efforts.

### Genome Size

**Figure 5:**
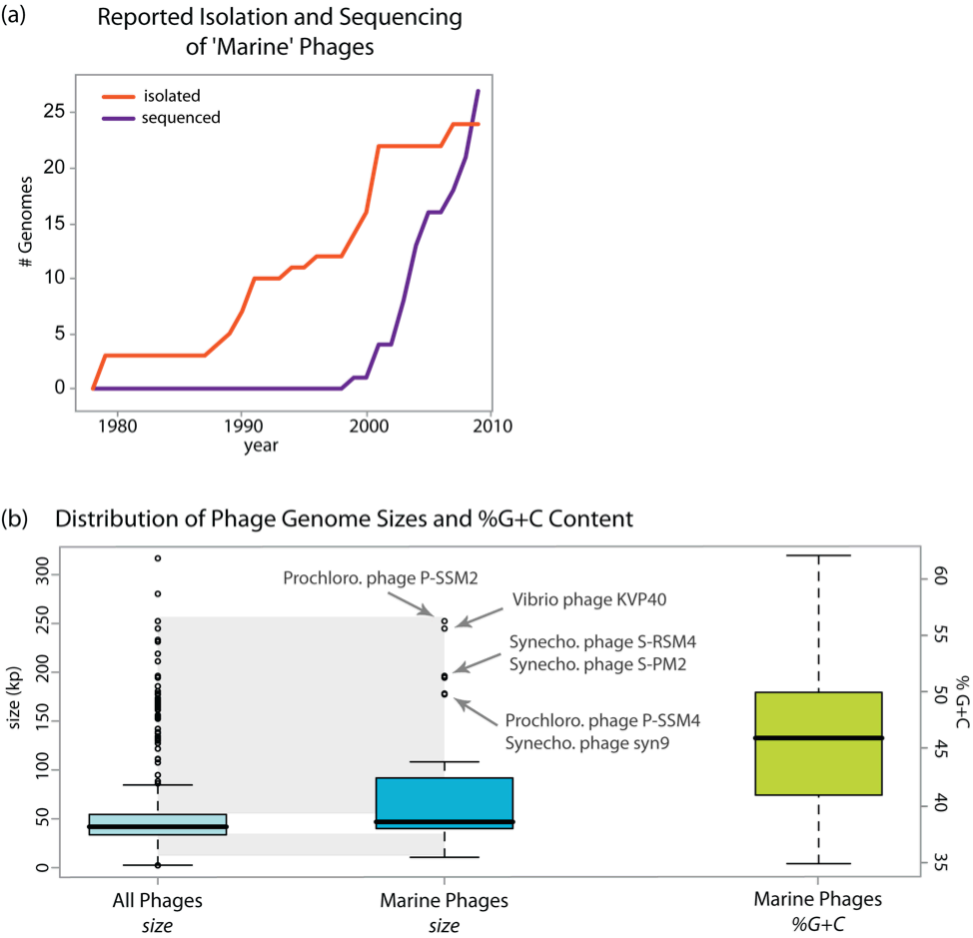
Overview of marine phage isolation, sequencing year, and genome properties stored in GCDML reports. (a) Trends of isolation and sequencing of the sequenced ‘marine’ phages over the last two decades. (b) Box and whisker plots showing range and distribution of genome sizes for all versus marine phages and %G+C content for marine phages. The box shows the interquartile range (middle 50% of the data); the thick black line demarcates the median, the dotted line extends to the minimum and maximum values; outliers are shown by empty circles. Data for genome sizes of “All Phages” were retrieved from NCBI.

Genome size has been implicated as diagnostic of biological properties of the phage; size is directly correlated with virion complexity and interference with host cellular activities [26]. Based on genome size, one-third of the sequenced marine phages are in the 75th percentile of all sequenced phages (Figure 5). As we sequence more phage genomes, it appears that those of marine phage are generally among the largest known [3,5] (Panel b of Figure 5). In the future, a closer look at the gene content of marine *vs.* non-marine phages could suggest whether this size is due to the great number of host-related genes carried by marine phages [2-6], or some other underlying evolutionary process.

### Taxonomic Diversity

**Figure 6:**
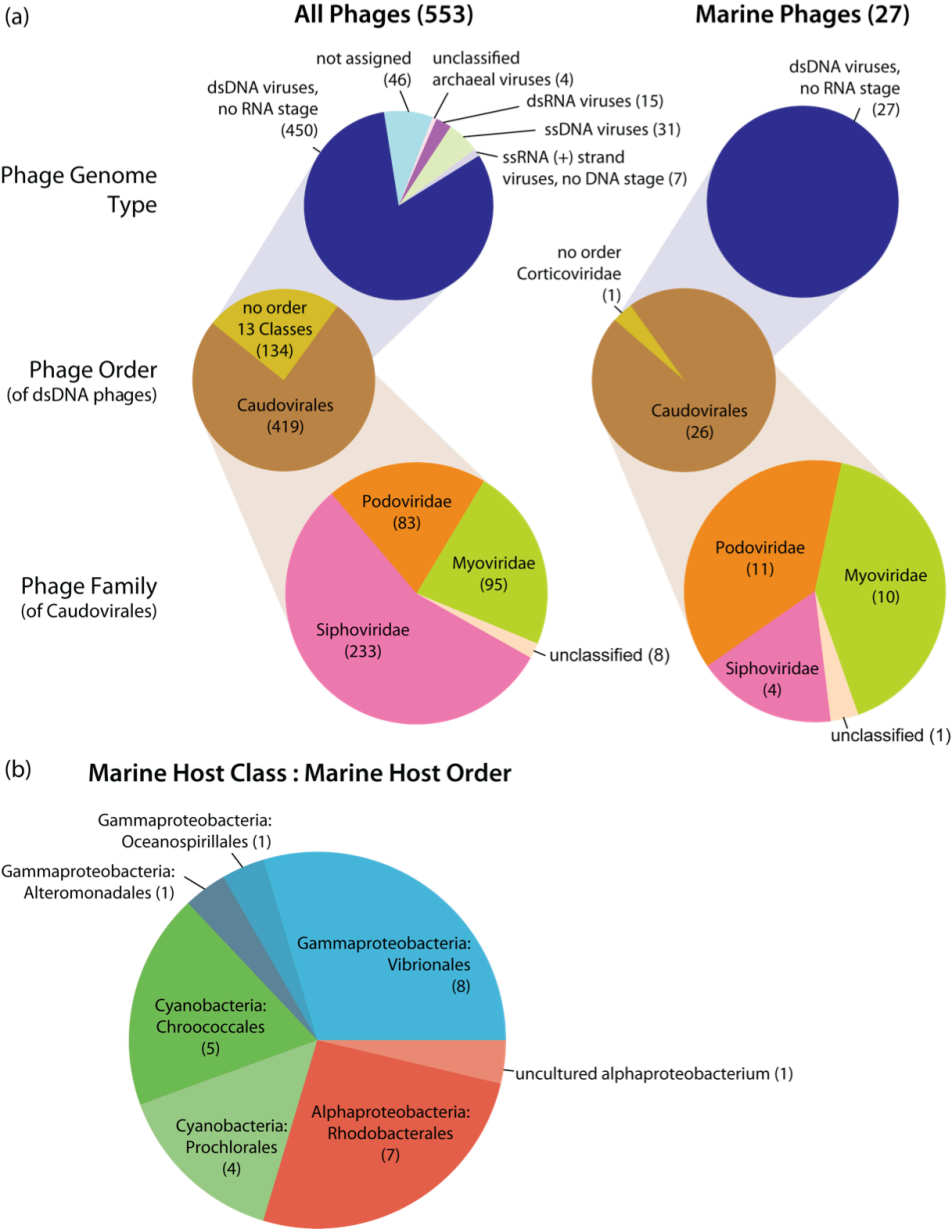
Overview of phage taxonomic data. (a)The taxonomic distribution of all sequenced phages versus all sequenced marine phages and (b) the hosts of all sequenced marine phages. All information describing marine phages and their hosts is accessible via GCDML reports.

The taxonomic diversity of sequenced marine phages is quite low as compared to the diversity of the sequenced phages from all habitats (Figure 6). Of the 27 marine phages sequenced, all are double-stranded DNA phages, with no RNA stage; 96% are of the viral order *Caudovirales* (*Pseudoalteromonas* phage PM2 has an unclassified order and belongs to the *Corticoviridae* family), as opposed to 76% of all sequenced phages (123 phages with no order span 13 different Classes).

Among all sequenced phages, there is general bias towards double-stranded DNA (dsDNA) viruses lacking an RNA stage (possibly influenced by, e.g., cloning biases in sequencing efforts, chloroform extractions that disrupt lipid-membranes of, i.e., dsRNA viruses, the difficulty in culturing archaeal hosts, etc.), despite the fact that, from an epidemiological perspective, over 75% of all viral diseases are the result of RNA viruses [27], which are yet to be represented by any sequenced marine phage isolates. The odd dsRNA phages have segmented genomes, whereby multiple 'chromosomes' exist in each virion and are often re-assorted during co-infection of the same host [28], where phages can exist in a 'carrier state', reproducing without killing their host [29]. This feature, combined with the intrinsic low fidelity of RNA replication, allows for RNA viruses to rapidly adapt to new environments, offering insights into modeling of viral population genetics and evolutionary theory that we can not yet consider in the marine realm [27]. ssDNA phages are also one of the major 'odd' phages groups not yet represented in the marine phage genome collection (Panel a of Figure 6), and are also under selective pressure quite unique from their dsDNA counterparts [30].

### Distribution of hosts

The distribution of their hosts is also biased (Figures 4 and 5). Two thirds of the sequenced marine phages infect *Proteobacteria*. Furthermore, most hosts are restricted to three major sets; 30% infect *Vibrio* spp., 33% infect Cyanobacteria (either *Chroococcales* or *Prochlorales*), and another 30% infect *Alphaproteobacteria* (all but one infect *Rhodobacterales*) (Panel b of Figure 6). All sequenced marine phages infect only two of the twenty-four *Bacteria* phyla (*Proteobacteria* and *Cyanobacteria*) and no *Archaea* (Panel b of Figure 6). Of these, only four families are represented, which also reflects metabolic/niche biases towards interest in: pathogenicity (namely phages of *Vibrio parahaemolyticus* infecting the *Vibrionales*), marine phototrophs (*Chroococcales* and *Prochlorales*), and ubiquitous, easily culturable coastal microbes essential to global carbon and sulfur cycles (*Rhodobacterales*) [31]. A similar pattern of habitat-driven taxonomic bias was seen in the first ecogenomic survey of sequenced microbial genomes, whereby 67% of the sequenced marine microbes were phototrophs [8].

### Genome Pairs

**Figure 7:**
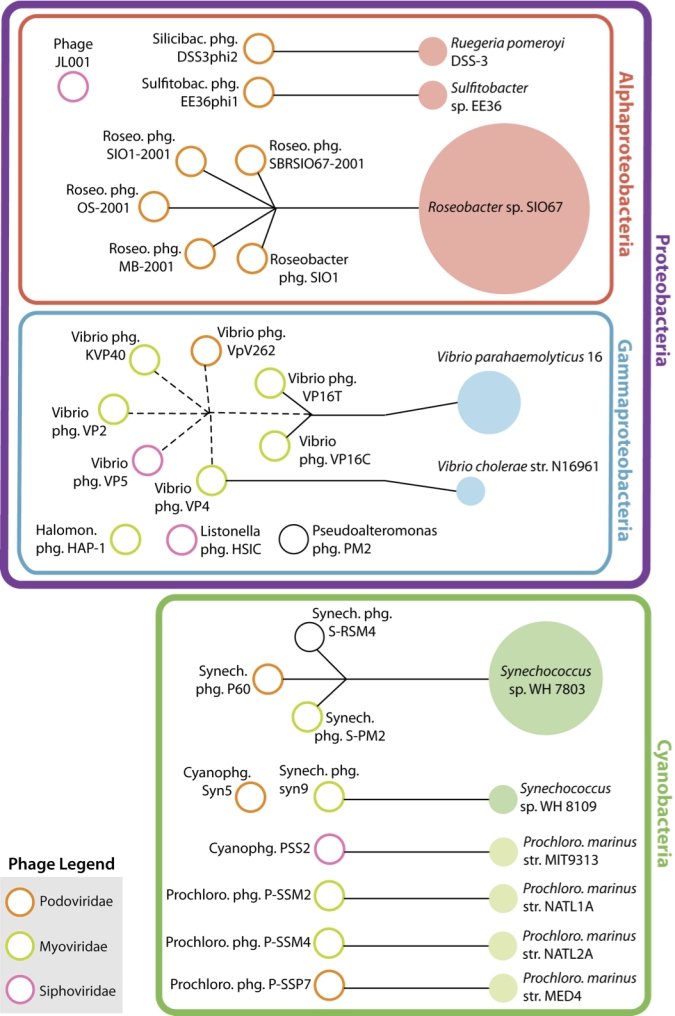
Network of 'genome pairs' and interactions between sequenced marine phages and sequenced hosts. Solid lines link phages (empty circles) to the host strain (solid circles) they infect; dashed lines connect phages to the host species (but not necessarily strain) they infect. Phages with no sequenced host are grouped by host Class (or Subclass for Cyanobacteria). Phage taxonomy is reflected by the color of the empty phage circle. Number of phages infecting a sequenced host is reflected by the size of the solid host circles.

The study of phages and hosts intrinsically lends itself to taking advantage of what Martiny and Field describe as "one of the most exciting and underutilized aspects of the genome collection" [8]: genome pairs. A genome pair occurs when organisms with potential natural interactions are both sequenced, e.g., a phage and host. These associations have revealed patterns in genome biology, such as how well pairs correlate based on %G+C content or tetranucleotide genome signatures [8,32]. Such pairs can (and soon will) rapidly evolve to complex networks as multiple phages infecting the same host, or multiple hosts infected by the same phage, are sequenced. This complexity obviates the need for the basic units, the pairs, to be explicitly documented (as called for by MIGS) in a structured form. This is possible through the GCDML 'original host' and 'alternate host' fields, where they can be stored for automated retrieval and network visualization. This process was just barely possible by hand with the 27 marine phage genomes, and reveals interesting trends (Figure 7). Thus far, most cyanophage-cyanobacteria associations are one-to-one pairs, though many cyanophages are known with broad host ranges [33]. Furthermore, such visualization leads to hypotheses about the 'lone phages', such as Phage phiJL001, *Halomonas* phage HAP-1, and Cyanophage Syn5, which lack a sequenced host, but which exist in phylogenetic groups with related sequenced hosts (Figure 7). The current map is useful in designing future sequencing ventures to answer targeted questions, such as "What drives phage host range” and “what are the genomic consequences of all members belonging to the same network?"

### Environmental parameters

Additionally, the 27 ‘mappable’ genomes can be further analyzed in their environmental context using emerging resources, such as megx.net, to (i) ‘put them on the map’ (Panel a of Figure 4; [14]), and (ii) extract interpolated environmental data, though only possible for the eight genomes where depth is reported and which are not too close to the coast (Table 1). Preliminary analysis of the megx.net interpolated data available in the GCDML reports revealed that, based on physical-chemical parameters across sample sites, e.g., the four phages isolated from the Sargasso Sea cluster together, while Cyanophage PSS2 appears to be an outlier (Panel b of Figure 4). Further examination of the range and distribution of each parameter show the Cyanophage PSS2 sample site to have quite distinct interpolated nitrate, phosphate, and dissolved oxygen values (Panel c of Figure 4).

The lack of explicit sample site geographic location and time (*x*, *y*, *z*, *t*) is apparent (Figure 3), and for environmental isolates, this may be the most 'value-added' component of MIGS compliance. These elements allow for genomes to be "put on the map" [20], thus reaping the benefits of, for example, comparisons using environmental data, either collected *in situ*, or interpolated using, i.e., the megx.net GIS Tools [16].

Using the resources of megx.net, any sample site in the ocean where location, depth, and time (*x*, *y*, *t*, *z*) are known can be supplemented by interpolated environmental data, such as temperature, salinity, phosphate, silicate, nitrate, dissolved oxygen, Apparent Oxygen Utilization (AOU), oxygen saturation, and chlorophyll, at standard depth levels for various time periods [16]. Geo-referenced genomes can be viewed in their environmental context on a world map (Panel a of Figure 4), and can be overlaid on numerous map data layers, such as nitrate, phosphate, silicate, and chlorophyll, or the environmental stability (expressed as standard deviations) of a parameter. Having such environmental data easily accessible and integrated with sequenced entities via GCDML reports allows for a rapid, automated "first pass" evaluation of environmental/ecological clusters and outliers (Panels b and c of Figure 4). This process greatly facilitates hypothesis and research question generation, such as: "what are the functional implications of Cyanophage PSS2 being isolated from such a comparatively high nutrient, low oxygen site?" and "what genomic features might be shared among isolates from similar habitats, such as the Sargasso Sea cluster?" Having such data accessible narrows the search time and space as researchers design comparative genomic, and even laboratory, studies.

## Discussion

We have manually curated MIGS-compliant GCDML reports for the 30 sequenced marine phage genomes currently available (Figure 1 and Figure 3).This study (i) is the first to publish a set of legacy MIGS reports for public genomes, (ii) is the first to publish MIGS reports for phage, and (iii) helps to establish ecogenomic trends within the sequenced marine phage genome collection using contextual data, with the end-goal of capturing richer descriptions of our public collection of genomes [8].

### Towards consistency and persistence of contextual data

This work shows that MIGS-compliant fields are largely missing for legacy genomes. This study found the most overlooked components to be sample site location (*x*, *y*, *z*), sample collection date (*t*), host range, and whether the organism exists in a culture collection (Figure 3). Likewise, nearly all of the 'Sequencing' components (Figure 3) are missing or filled with a 'not available' placeholder in the final MIGS reports, even following curation. In a world of rapidly evolving technologies, this component is critical as techniques change through time.

Implementing standards, such as those of the GSC, is an invaluable means to encourage sequence submitters to carry contextual data over to the public databases. As nearly 60% of the data missing from INSDC reports needed to be supplemented by manual curation (Figure 3), it is not the case that this data is too difficult to collect or that MIGS is not possible to comply with. Through these efforts to collect richer contextual data, we can better highlight gaps in our biological knowledge of marine phage, and use contextual data to establish "rules and exceptions" [8] to describe the impact of viruses in the marine realm.
